# Orexigenic Hormone Ghrelin Attenuates Local and Remote Organ Injury after Intestinal Ischemia-Reperfusion

**DOI:** 10.1371/journal.pone.0002026

**Published:** 2008-04-23

**Authors:** Rongqian Wu, Weifeng Dong, Youxin Ji, Mian Zhou, Corrado P. Marini, Thanjavur S. Ravikumar, Ping Wang

**Affiliations:** 1 The Feinstein Institute for Medical Research, Manhasset, New York, United States of America; 2 Department of Surgery, North Shore University Hospital and Long Island Jewish Medical Center, Manhasset, New York, United States of America; Oregon Health & Science University, United States of America

## Abstract

**Background:**

Gut ischemia/reperfusion (I/R) injury is a serious condition in intensive care patients. Activation of immune cells adjacent to the huge endothelial cell surface area of the intestinal microvasculature produces initially local and then systemic inflammatory responses. Stimulation of the vagus nerve can rapidly attenuate systemic inflammatory responses through inhibiting the activation of macrophages and endothelial cells. Ghrelin, a novel orexigenic hormone, is produced predominately in the gastrointestinal system. Ghrelin receptors are expressed at a high density in the dorsal vagal complex of the brain stem. In this study, we investigated the regulation of the cholinergic anti-inflammatory pathway by the novel gastrointestinal hormone, ghrelin, after gut I/R.

**Methods and Findings:**

Gut ischemia was induced by placing a microvascular clip across the superior mesenteric artery for 90 min in male adult rats. Our results showed that ghrelin levels were significantly reduced after gut I/R and that ghrelin administration inhibited pro-inflammatory cytokine release, reduced neutrophil infiltration, ameliorated intestinal barrier dysfunction, attenuated organ injury, and improved survival after gut I/R. Administration of a specific ghrelin receptor antagonist worsened gut I/R-induced organ injury and mortality. To determine whether ghrelin's beneficial effects after gut I/R require the intact vagus nerve, vagotomy was performed in sham and gut I/R animals immediately prior to the induction of gut ischemia. Our result showed that vagotomy completely eliminated ghrelin's beneficial effect after gut I/R. To further confirm that ghrelin's beneficial effects after gut I/R are mediated through the central nervous system, intracerebroventricular administration of ghrelin was performed at the beginning of reperfusion after 90-min gut ischemia. Our result showed that intracerebroventricular injection of ghrelin also protected the rats from gut I/R injury.

**Conclusions:**

These findings suggest that ghrelin attenuates excessive inflammation and reduces organ injury after gut I/R through activation of the cholinergic anti-inflammatory pathway.

## Introduction

Acute mesenteric ischemia is an abdominal emergency with a mortality rate of up to 60–80% [1], [2]. The intestinal mucosa is extremely susceptible to ischemia/reperfusion (I/R) injury. Even short periods of ischemia can induce the systemic production of various inflammatory mediators and activates leukocytes, which may lead to remote organ injury and subsequent mortality. The vagus nerve has been shown to convey the immunologic state of the gastrointestinal tract to the brain [3]. Activation of the vagus nerve during systemic stress confers a protective advantage to the host by restraining a potentially adverse peripheral immune response. This physiological mechanism, termed the cholinergic anti-inflammatory pathway, modulates host inflammatory responses via cholinergic mediators or by electrical stimulation of the vagus nerve [4]–[6].

Ghrelin, an orexigenic hormone, was first identified in 1999 as an endogenous ligand for the growth hormone secretagogue receptor type 1a (GHSR-1a, i.e., ghrelin receptor) [7]. Ghrelin is found throughout the gastrointestinal tract [8]. In addition to its growth hormone-releasing properties [9], ghrelin has now been proved to possess other endocrine and non-endocrine activities reflecting central and peripheral GHSR-1a distribution [10], [11]. Administration of ghrelin is beneficial following heart failure or cardiac ischemia/reperfusion injury [12], [13]. However, the precise mechanism responsible for these beneficial effects remains largely unknown. Our recent study has demonstrated that ghrelin's direct effect on inflammatory cytokine release from macrophages is negligible [14]. Since intracerebroventricular (ICV) injection of ghrelin stimulates the efferent vagus nerve [15], it has been postulated that the anti-inflammatory property of ghrelin is mediated through vagus nerve stimulation [11]. However, it remains unknown whether ghrelin has any protective effects on gut I/R-induced local and remote organ injury and, if so, the potential mechanism responsible for it. This study was conducted to test the hypothesis that ghrelin attenuates gut I/R-induced local and remote organ injury and mortality through the activation of the cholinergic anti-inflammatory pathway.

## Results

### Ghrelin levels decrease after gut I/R

**Figure 1:**
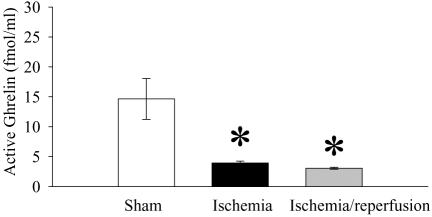
Alterations in plasma levels of ghrelin at the end of 90-min gut ischemia (Ischemia), 2 h reperfusion after ischemia (Ischemia/reperfusion), or sham operation (Sham). Data are presented as means±SE (n = 5–6) and compared by one-way ANOVA and Student-Newman-Keuls test: * *P*<0.05 versus Sham group.

As shown in Figure 1, plasma levels of ghrelin in sham operated animals were 14.6 fmol/ml. At the end of 90 min ischemia, plasma levels of ghrelin decreased by 73% (P<0.05). A slight further decrease in plasma levels of ghrelin was found at 2 h after reperfusion. However, there was no statistically significant difference in plasma levels of ghrelin between ischemia alone and I/R animals.

### Ghrelin inhibits proinflammatory responses after gut I/R

**Figure 2:**
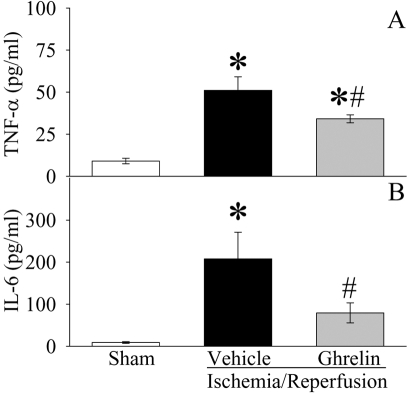
Alterations in serum levels of TNF-α (A) and IL-6 (B) in sham-operated animals (Sham) and ischemia/reperfusion animals treated with normal saline (Vehicle) or ghrelin (Ghrelin) at 1.5 h after the completion of treatment (i.e., 2 h after reperfusion). Data are presented as means±SE (n = 6/group) and compared by one-way analysis of variance (ANOVA) and Student-Newman-Keuls test: *P<0.05 versus Sham group; #*P*<0.05 versus I/R+Vehicle group.

**Figure 3:**
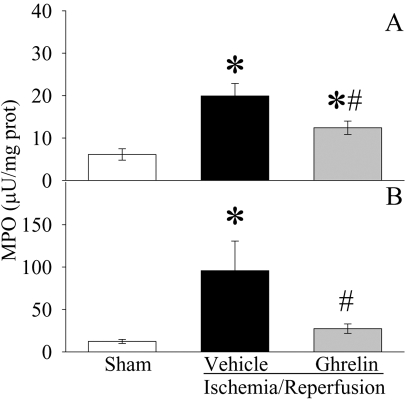
Alterations in intestinal (A) and pulmonary (B) MPO activities in sham-operated animals (Sham) and ischemia/reperfusion animals treated with normal saline (Vehicle) or ghrelin (Ghrelin) at 1.5 h after the completion of treatment (i.e., 2 h after reperfusion). Data are presented as means±SE (n = 5–6) and compared by one-way ANOVA and Student-Newman-Keuls test: * *P*<0.05 versus Sham group; # *P*<0.05 versus Vehicle group.

As indicated in Figure 2A, serum levels of TNF-α increased by 4.7 fold at 2 h after gut I/R (P<0.05). Treatment with ghrelin reduced serum TNF-α levels by 33% in gut I/R animals (P<0.05, Fig. 2A). Similarly, serum levels of IL-6 increased by 22 fold 2 h after gut I/R (P<0.05, Fig. 2B). Ghrelin treatment decreased them by 62% (P<0.05), and there was no significant difference in serum IL-6 levels between sham-operated and ghrelin treated gut I/R animals (Fig. 2B). The level of myeloperoxidase (MPO) activity is an indicator of neutrophil infiltration. As demonstrated in Figures 3A-B, gut I/R induced a significant increase in intestinal and pulmonary MPO activities in vehicle-treated rats as compared with sham animals. Treatment with ghrelin significantly inhibited the increase in both intestinal (Fig. 3A) and pulmonary (Fig. 3B) MPO activities after gut I/R (P<0.05). These results show that ghrelin downregulates proinflammatory cytokines and attenuates the influx of neutrophils into the gut and lungs after gut I/R.

### Ghrelin ameliorates intestinal barrier dysfunction after gut I/R

**Figure 4:**
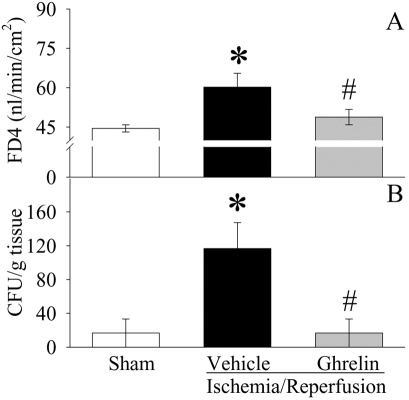
Alterations in intestinal mucosal permeability (A) to fluorescein isothiocyanate dextran with a molecular weight of 4000 Da (FD4) and bacterial translocation to mesenteric lymph nodes (B) in sham-operated animals (Sham) and ischemia/reperfusion animals treated with normal saline (Vehicle) or ghrelin (Ghrelin) at 1.5 h after the completion of treatment (i.e., 2 h after reperfusion). Data are presented as means±SE (n = 6), and compared by one-way ANOVA and Student-Newman-Keuls test: * P<0.05 versus Sham group; # P<0.05 versus Vehicle group.

Intestinal barrier dysfunction, manifested by increased mucosal permeability to hydrophilic macromolecules and/or increased bacterial translocation to mesenteric lymph nodes, occurs following gut I/R [16]. As indicated in Figure 4A, ileal mucosal permeability to the fluorescent macromolecule, FD4, was significantly increased at 2 h after reperfusion in vehicle treated animals as compared with sham controls (P<0.05). Similarly, bacterial translocation to mesenteric lymph nodes was minimal in the sham group, but was extensive in the gut I/R vehicle treated group (P<0.05, Fig. 4B). Treatment with ghrelin at the time of reperfusion, however, significantly ameliorated the development of both ileal mucosal hyperpermeability and bacterial translocation.

### Ghrelin attenuates organ injury after gut I/R

**Figure 5:**
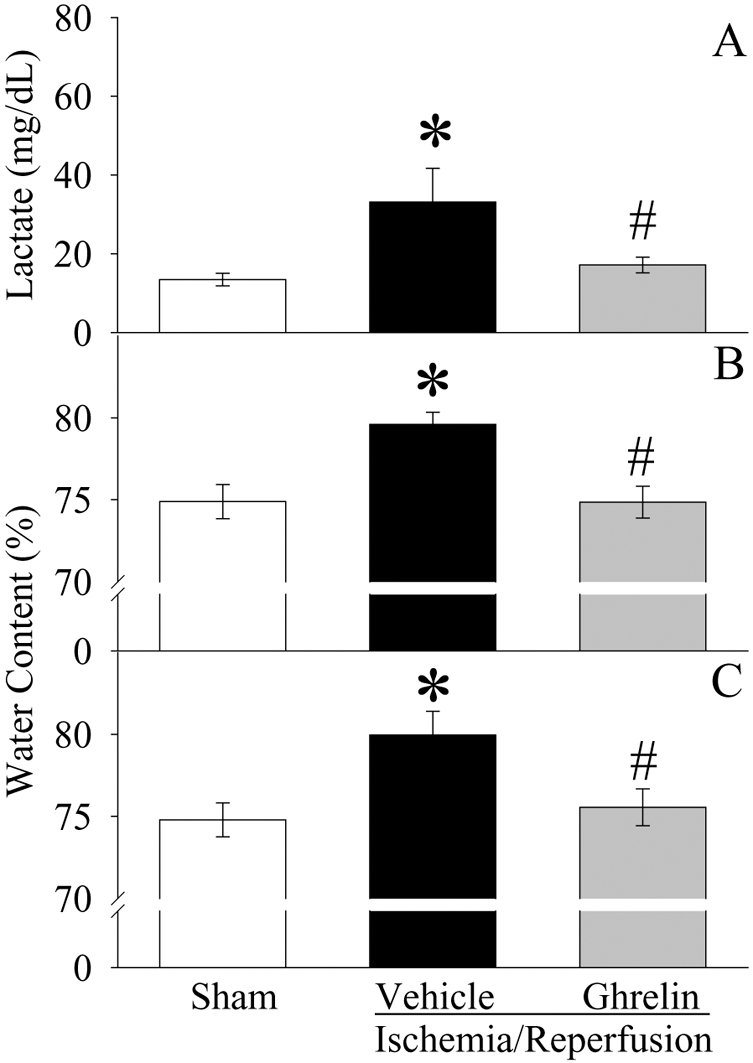
Alterations in serum levels of lactate (A), gut (B) and lung (C) water content in sham-operated animals (Sham) and ischemia/reperfusion animals treated with normal saline (Vehicle) or ghrelin (Ghrelin) at 1.5 h after the completion of treatment (i.e., 2 h after reperfusion). Data are presented as means±SE (n = 5–6) and compared by one-way ANOVA and Student-Newman-Keuls test: * *P*<0.05 versus Sham group; # *P*<0.05 versus Vehicle group.

**Figure 6:**
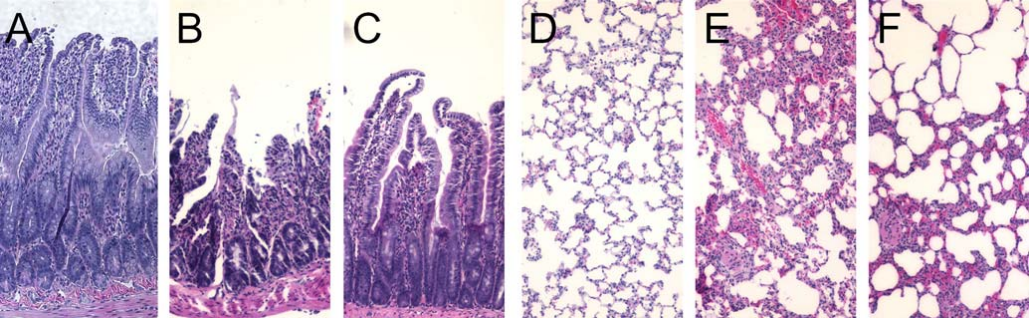
Alterations in morphologic photomicrography of the small intestine and lungs. A, a photomicrograph of a small intestinal section from a sham-operated rat; B, a photomicrograph of a small intestinal section from an I/R rat at 2 h after gut ischemia-reperfusion treated with vehicle; C, a photomicrograph of a small intestinal section from an I/R rat at 2 h after gut ischemia-reperfusion treated with ghrelin; D, a photomicrography of a pulmonary section from a sham-operated rat. E, a photomicrography of lung section from an I/R rat at 2 h after gut ischemia-reperfusion treated with vehicle; F, a photomicrography of lung section from an I/R rat at 2 h after gut ischemia-reperfusion treated with ghrelin. Original magnification, ×200.

Compared with sham operated animals, serum levels of lactate were more than doubled at 2 h after gut I/R in vehicle treated animals (P<0.05, Fig. 5A). Ghrelin treatment significantly reduced serum levels of lactate by 48% (P<0.05; Fig. 5A). As shown in Figures 5B–C, rats subjected to gut I/R had a significant increase in gut and lung water content as compared with sham-operated animals (P<0.05). When I/R animals were treated with ghrelin, the water content of the gut (Fig. 5B) and lungs (Fig. 5C) was reduced and there was no statistical difference in gut and lung water content between sham-operated and I/R ghrelin treated animals. In terms of the histopathological changes, mucosal destruction, loss of villi and epithelial cells, hemorrhage, and infiltration of inflammatory cells were observed microscopically in the rat intestine after I/R (Fig. 6B) as compared with sham controls (Fig. 6A). Treatment with ghrelin dramatically improved these microscopic alterations (Fig. 6C). Similarly, lung injury characterized by disruption of lung architecture, extravasation of red blood cells, and accumulation of inflammatory cells was presented in I/R-vehicle treated animals (Fig. 6E). Ghrelin treated rats revealed a marked reduction in infiltrated inflammatory cells and improvement of lung architecture (Fig. 6F).

### Ghrelin improves survival after gut I/R

**Figure 7:**
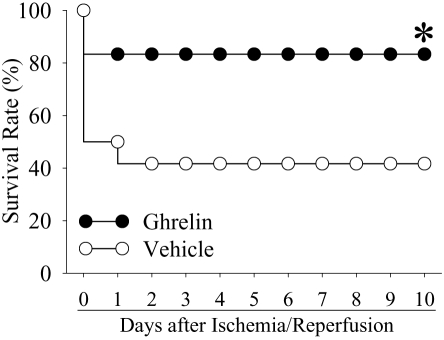
Alterations in the survival rate at 10 days after gut ischemia/reperfusion with normal saline treatment (Vehicle) and gut ischemia/reperfusion with ghrelin treatment (Ghrelin). Ghrelin (12 nmol/kg BW), or vehicle (1-ml normal saline) was administered intravenously over a period of 30 min to animals that underwent 90 min gut ischemia immediately after removing the microvascular clip. The animals were then allowed food and water *ad libitum* and were monitored for 10 days to record survival. There were 12 animals in each group. The survival rate was estimated by the Kaplan-Meier method and compared by using the log-rank test. * *P*<0.05 vs. Vehicle.

The survival rate after gut I/R with vehicle administration was 50% at day 1 and decreased to 41.6% at days 2–10 (Fig. 7). Treatment with ghrelin, however, improved the survival rate to 83.3%, which was significantly higher than that in the gut I/R vehicle treated animals (P<0.05, Fig. 7).

### Ghrelin receptor blockade exacerbates organ injury and increases mortality after gut I/R

**Figure 8:**
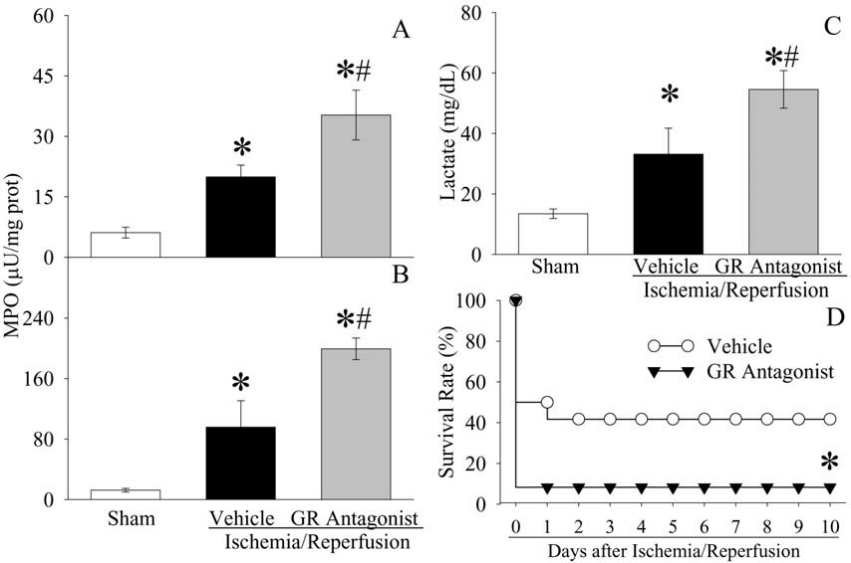
Alterations in intestinal (A) and pulmonary (B) MPO activities, and serum levels of lactate (C) in sham-operated animals (Sham) and ischemia/reperfusion animals treated with normal saline (Vehicle) or [D-Arg^1^ D-Phe^5^ D-Trp^7, 9^ Leu^11^]-substance P (GA antagonist) at 1.5 h after the completion of treatment (i.e., 2 h after reperfusion). Data are presented as means±SE (n = 5–6) and compared by one-way ANOVA and Student-Newman-Keuls test: * *P*<0.05 versus Sham group; # *P*<0.05 versus Vehicle group. Alterations in the survival rate (D) at 10 days after gut ischemia/reperfusion with normal saline treatment (Vehicle) and gut ischemia/reperfusion with [D-Arg^1^ D-Phe^5^ D-Trp^7, 9^ Leu^11^]-substance P (GA antagonist). There were 12 animals in each group. Survival rate in the vehicle treated animals is derived from Figure 7. The survival rate was estimated by the Kaplan-Meier method and compared by using the log-rank test. * *P*<0.05 vs. Vehicle.

To further define the role of ghrelin deficiency in gut I/R injury, [D-Arg^1^ D-Phe^5^ D-Trp^7, 9^ Leu^11^]-substance P was administered to gut I/R animals. As shown in Figures 8A–B, neutrophil infiltration was further increased after [D-Arg^1^ D-Phe^5^ D-Trp^7, 9^ Leu^11^]-substance P administration in gut I/R animals as demonstrated by elevated levels of MPO activities in both gut and lungs as compared with those in gut I/R vehicle-treated animals (P<0.05). Similarly, [D-Arg^1^ D-Phe^5^ D-Trp^7, 9^ Leu^11^]-substance P further increased serum levels of lactate by 65% (P<0.05, Fig. 8C). Most importantly, the 10-day survival rate after gut I/R was decreased to 8.3% by [D-Arg^1^ D-Phe^5^ D-Trp^7, 9^ Leu^11^]-substance P administration (P<0.05, Fig. 8D).

### Ghrelin's beneficial effects after gut I/R require the intact vagus nerve

**Figure 9:**
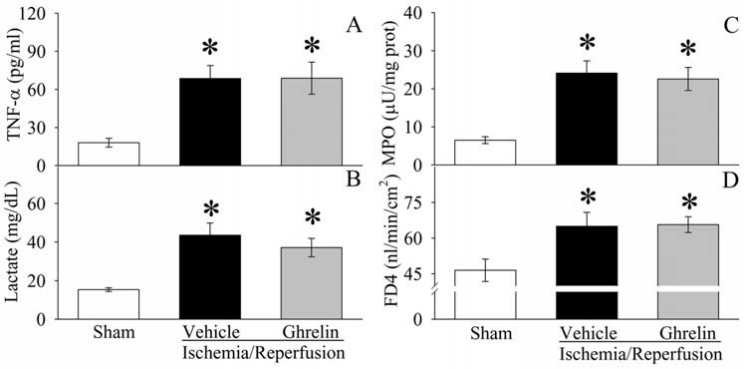
Effects of vagotyomy on serum levels of TNF-α (A) and lactate (B), intestinal MPO activities (C), and intestinal mucosal permeability (D) to fluorescein isothiocyanate dextran with a molecular weight of 4000 Da (FD4) in sham-operated animals (Sham) and ischemia/reperfusion animals treated with normal saline (Vehicle) or ghrelin (Ghrelin) at 1.5 h after the completion of treatment (i.e., 2 h after reperfusion). Data are expressed as means±SE (n = 6–7/group) and compared by one-way analysis of variance (ANOVA) and Student-Newman-Keuls test: *P<0.05 versus sham-operated animals.

To determine whether ghrelin's beneficial effects after gut I/R require the intact vagus nerve, vagotomy was performed in sham and gut I/R animals immediately prior to the induction of gut ischemia as we described recently [14]. As indicated in Figures 9A–B, vagotomy completely eliminates the effect of this agent on serum levels of TNF-α and lactate. Similarly, ghrelin's effects on neutrophil infiltration in the gut (Fig. 9C) and intestinal barrier dysfunction (Fig. 9D) after gut I/R are also completely abolished by vagotomy. Thus, ghrelin's beneficial effects after gut I/R require the intact vagus nerve.

### Intracerebroventricular (ICV) administration of ghrelin is protective after gut I/R

**Figure 10:**
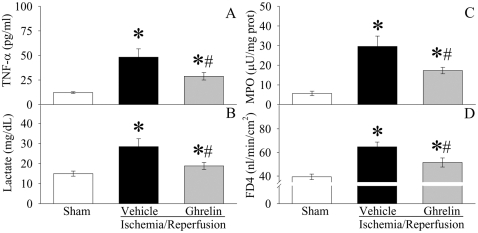
Alterations in serum levels of TNF-α (A) and lactate (B), intestinal MPO activities (C), and intestinal mucosal permeability (D) to fluorescein isothiocyanate dextran with a molecular weight of 4000 Da (FD4) in sham-operated animals (Sham) and ischemia/reperfusion animals treated with intracerebroventricular (ICV) injection of normal saline (Vehicle) or ghrelin (Ghrelin) at 1.5 h after the completion of treatment (i.e., 2 h after reperfusion). Data are presented as means±SE (n = 6) and compared by one-way ANOVA and Student-Newman-Keuls test: * *P*<0.05 versus Sham group; # *P*<0.05 versus Vehicle group.

To further confirm that ghrelin's beneficial effects after gut I/R are mediated through the central nervous system, ICV administration of ghrelin was performed at the beginning of reperfusion after 90-min gut ischemia. As shown in Figures 10A–B, ICV injection of ghrelin significantly inhibits TNF-α release (P<0.05, Fig. 10A), reduces serum levels of lactate (P<0.05, Fig. 10B). Similarly, ICV injection of ghrelin also decreases neutrophil infiltration in the gut (P<0.05, Fig. 10C) and ameliorates intestinal barrier dysfunction (P<0.05, Fig. 10D).

## Discussion

Gut I/R injury is a serious condition in intensive care patients. Activation of immune cells adjacent to the huge endothelial cell surface area of the intestinal microvasculature produces initially local and then systemic inflammatory responses. Balanced inflammatory responses are essential elements of a successful host response after injury. However, excessive and sustained inflammatory responses can lead to severe tissue damage. Using an established animal model of gut I/R (i.e., superior mesenteric artery occlusion), the present study shows that plasma levels of ghrelin are significantly reduced after gut I/R. Therefore, there is a ghrelin deficiency after gut I/R. Ghrelin is a novel gastrointestinal hormone. The biological effects of ghrelin are mediated through the ghrelin receptor (i.e., GHSR-1a), a 7 transmembrane domain Gq protein coupled receptor [7], [17]. Ghrelin is the only identified endogenous ligand for this receptor. Ghrelin was originally reported to induce growth hormone release through pituitary GHSR-1a stimulation [9], [18], [19]. However, a large body of evidence has indicated other physiological functions of ghrelin mediated by the central and peripheral ghrelin receptors [10]. GHSR-1a is found in the brain stem, pituitary, hypothalamus, heart, blood vessels, lungs, stomach, pancreas, intestines, kidneys, and adipose tissue [20]–[22]. The wide distribution of GHSR-1a suggests multiple paracrine, autocrine and endocrine roles of ghrelin [20]–[23]. It has been linked to the regulation of pituitary hormone secretion, feeding, energy homeostasis, gastrointestinal function, and cardiovascular and immune system [14], [24], [25]. Since administration of a specific ghrelin receptor antagonist increases proinflammatory cytokine release in normal animals [26], [27], the acute ghrelin deficiency after gut I/R may contribute to local and systemic inflammatory derangements under such conditions. The result, that the ghrelin receptor antagonist worsens gut I/R-induced organ injury and mortality, further confirms the important role of ghrelin deficiency in gut I/R injury.

Our current results also show that ghrelin administration inhibits proinflammatory cytokine release, reduces neutrophil infiltration, ameliorates intestinal barrier dysfunction, attenuates organ injury, and improves survival after gut I/R. However, ghrelin's direct effect on inflammatory cytokine release from macrophages is negligible [14]. It has been reported that ghrelin activates the vagus nerve and vagal blockade abolishes ghrelin-induced feeding and growth hormone secretion [28]. The current study also indicates that the protection of ghrelin after gut I/R requires the intact vagus nerve, as vagotomy prevents its beneficial effects. Ghrelin can cross the blood-brain barrier [29]–[31]. Since ICV injection of ghrelin is also protective after gut I/R, it appears that the stimulatory effect of ghrelin on the vagus nerve is mainly mediated via the central nervous system. This is not surprising, because ghrelin receptors are expressed at a high density in the brain [22], [32]. Stimulation of the vagus nerve can rapidly attenuate systemic inflammatory responses through inhibiting the activation of macrophages and endothelial cells. This physiological mechanism, termed ‘the cholinergic anti-inflammatory pathway’, can reflexively monitor and adjust the inflammatory response to prevent excessive inflammation. This pathway has major implications in immunology; however, the endogenous molecule(s) for the regulation of this pathway remains unknown. Our current findings strongly suggest that ghrelin may be the circulating mediator for the link between the vagus nerve and the immune system.

An underlying defect in the activity of the cholinergic anti-inflammatory pathway may trigger an overwhelming and self-destructive immune response to an otherwise innocuous immunological stimulus and thus contribute to disease pathogenesis [3], [33]. Abundant evidence points to the concept that complex behavior originating in higher brain centers can directly influence outflow through the vagus nerve. Sudden death, increased morbidity and mortality following cardiac surgery in hostile or depressed patients, and increased death rates in patients with sepsis or organ failure can be linked clinically to decreased vagus nerve activity [34]–[38]. Although it is not currently known whether vagus nerve activity in the heart correlates with the activation of the cholinergic anti-inflammatory pathway, there have been reports of decreased cardiac vagus nerve activity in diseases associated with exaggerated inflammatory responses [33]. Clinical observations implicate a significant correlation between decreased instantaneous heart rate variability and increased morbidity and/or mortality in sepsis, rheumatoid arthritis, lupus, sarcoidosis, inflammatory bowel diseases, and trauma [39]–[42]. Thus, decreased circulating levels of ghrelin after gut I/R may impair the efficacy of the cholinergic anti-inflammatory pathway, thereby leading to the hyper-inflammatory state and subsequent local and remote organ injuries. These findings also suggest that administration of ghrelin after gut I/R attenuates excessive inflammation and reduces organ injury through the rapid activation of the cholinergic anti-inflammatory pathway. In this regard, ghrelin may be the therapeutic agent in various inflammatory disorders such as sepsis, hemorrhagic shock, and inflammatory bowel disease characterized by an overwhelming inflammatory response.

## Materials and Methods

### Animal model of gut ischemia/reperfusion

Male Sprague-Dawley rats (275–325g) were housed in a temperature-controlled room on a 12-h light/dark cycle and fed a standard Purina rat chow diet. Prior to the induction of ischemia, rats were fasted overnight but allowed water *ad libitum*. Rats were anesthetized with isoflurane inhalation and the ventral neck, abdomen and groin were shaved and washed with 10% povidone iodine. A catheter (PE-50 tubing) was placed in the femoral vein after carefully separating the femoral nerve and blood vessels. A 2-cm midline abdominal incision was performed. A microvascular clip was placed across the superior mesenteric artery (SMA) for 90 min. The microvascular clip was then removed to allow reperfusion, and the animals were randomly assigned to various groups. Sham animals (i.e., control animals) underwent the same surgical procedure with the exception of the SMA clamping. All experiments were performed in accordance with the National Institutes of Health guidelines for the use of experimental animals. This project was approved by the Institutional Animal Care and Use Committee of the Feinstein Institute for Medical Research.

### Intravenous administration of ghrelin

Immediately after removing the microvascular clip, ghrelin (12 nmol/kg BW, Phoenix Pharmaceuticals, Belmont, CA), or vehicle (1-ml normal saline) was administered intravenously over a period of 30 min through a pump (Harvard Apparatus, Holliston, MA). Animals were then anesthetized at 2 h after reperfusion (i.e., 1.5 h after the completion of treatment) or sham operation for collection of blood and tissue samples.

### Determination of plasma levels of ghrelin, TNF-α and IL-6

Plasma levels of ghrelin, TNF-α and IL-6 were quantified using an enzyme-linked immunosorbent assay (ELISA) kit specifically for ghrelin (Linco Research, Inc, St. Charles, MO), rat TNF-α or IL-6 (BD Biosciences, San Diego, CA). The assay was carried out according to the instructions provided by the manufacturer.

### Granulocyte myeloperoxidase assessment

Neutrophil accumulation within the gut and lung tissue was estimated using the MPO activity assay as we described previously [43].

### Water content determination and histological examination

Gut and lung edema were estimated by comparing tissue water content. Briefly, gut and lung tissues were dried in a 70°C oven for 48 h. Gut and lung water content was calculated as % H_2_O = (1−dry wt/wet wt) × 100%. The morphological alterations in the gut and lungs were examined by hematoxylin and eosin (H-E) staining.

### Determination of intestinal mucosal permeability

Intestinal barrier function was assessed by measuring translocation of the fluorescent tracer, fluorescein isothiocyanate dextran with a molecular weight of 4000 Da (FD4, Sigma) by the everted gut sac method as described by Fink *et al* [44]–[47] and recently used by us [48].

### Determination of bacterial translocation

The mesenteric lymph nodes complex was harvested and equal amount of wet tissues was homogenized and briefly centrifuged to remove gross particulate matters. Serial log dilutions of tissue homogenates were applied. Five hundred µl of each dilution was then plated on chocolate agar plates (Fisher Scientific) and incubated at 37°C for 24 h under aerobic conditions. The colony-forming units (CFU) were counted and results were expressed as CFU per gram of tissue.

### Determination of serum levels of lactate

Serum concentrations of lactate were determined by using an assay kit according to the manufacturer's instructions (Pointe Scientific, Lincoln Park, MI).

### Survival Study

In additional groups of animals, ghrelin (12 nmol/kg BW), or vehicle (1-ml normal saline) was administered intravenously over a period of 30 min to animals that underwent 90 min gut ischemia immediately after removing the microvascular clip as described above. The animals were then allowed food and water *ad libitum* and were monitored for 10 days to record survival.

### Administration of ghrelin receptor antagonist [D-Arg^1^ D-Phe^5^ D-Trp^7, 9^ Leu^11^]-substance P

To further define the role of ghrelin deficiency in gut I/R injury, a specific and potent ghrelin receptor antagonist, [D-Arg^1^ D-Phe^5^ D-Trp^7, 9^ Leu^11^]-substance P (Bachem, Torrance, CA) [49], was administered to intestinal I/R animals. Briefly, immediately after removing the microvascular clip, [D-Arg^1^ D-Phe^5^ D-Trp^7, 9^ Leu^11^]-substance P (0.2 µmol/kg BW in 1 ml normal saline) was administered intravenously over a period of 30 min through a pump. Serum levels of lactate, gut and lung MPO activity were determined at 2 h after reperfusion (i.e., 1.5 h after [D-Arg^1^ D-Phe^5^ D-Trp^7, 9^ Leu^11^]-substance P administration) or sham operation. In additional groups of animals, survival was recorded for 10 days thereafter.

### Vagotomy

The dorsal and ventral branches of the vagus nerve were dissected from the esophagus as we described recently [14]. Gut ischemia was then performed by SMA occlusion for 90 min. At the end of 90 min gut ischemia or sham operation, ghrelin or vehicle was administrated intravenously as described above in the vagus nerve intact animals. At 1.5 h after the completion of treatment or sham operation (i.e., 3.5 h after vagotomy), serum levels of TNF-α were measured by ELISA. Neutrophil infiltration in the gut was assessed by determining intestinal MPO activity. Organ injury was evaluated by measuring ileal mucosal permeability to FD4 and serum lactate levels.

### Intracerebroventricular (ICV) injection of ghrelin

To further confirm that ghrelin's beneficial effects after gut I/R are mediated through the central nervous system, ICV administration of ghrelin (1 nmol in 10 µl) was performed at the beginning of reperfusion after 90-min gut ischemia as we described recently [26]. At 2 h after reperfusion, serum levels of TNF-α were measured by ELISA. Neutrophil infiltration in the gut was assessed by determining MPO activity. Organ injury was evaluated by measuring ileal mucosal permeability to FD4 and serum lactate levels. The location of ICV injections was confirmed by histological examination of the brain after the experiment.

### Statistical analysis

All data are expressed as mean±standard error (SE) and compared by one-way analysis of variance (ANOVA) and Student-Newman-Keuls test for multiple group analysis. The survival rate was estimated by Kaplan-Meier method and compared by the log-rank test. Differences in values were considered significant if *P*<0.05.
